# Supplementary material for: Hybrid Models and Biological Model Reduction with PyDSTool
Source: PLoS Comput Biol. 2012 Aug 9;8(8):e1002628. doi: 10.1371/journal.pcbi.1002628 (PMC3415397; doi:10.1371/journal.pcbi.1002628)
Supplement: Text S4 — Complete source code for the PyDSTool package (version 0.88.120504). Includes API documentation and help files linking to web pages. This file is identical to the current public release on Sourceforge.net. (ZIP) [file pcbi.1002628.s004.zip › PyDSTool/html/PyDSTool.scipy_ode-module.html]

xml version="1.0" encoding="ascii"?


PyDSTool.scipy\_ode


| Home | Trees | Indices | Help | PyDSTool |
| --- | --- | --- | --- | --- |

Package PyDSTool ::
Module scipy\_ode

[hide private]

[frames] | no frames]

# Module scipy\_ode

source code

```
User-friendly interface to various numerical integrators for solving a
system of first order ODEs with prescribed initial conditions:

       d y(t)[i]
       ---------  = f(t,y(t))[i],
        d t

       y(t=0)[i] = y0[i],

where i = 0, ..., len(y0) - 1

Provides:
  ode  - a generic interface class to numeric integrators. It has the
         following methods:
           integrator = ode(f,jac=None)
           integrator = integrator.set_integrator(name,**params)
           integrator = integrator.set_initial_value(y0,t0=0.0)
           integrator = integrator.set_f_params(*args)
           integrator = integrator.set_jac_params(*args)
           y1 = integrator.integrate(t1,step=0,relax=0)
           flag = integrator.successful()

Supported integrators:
  vode - Variable-coefficient Ordinary Differential Equation solver,
         with fixed-leading-coefficient implementation.
         It provides implicit Adams method (for non-stiff problems)
         and a method based on backward differentiation formulas (BDF)
         (for stiff problems).
         Source: http://www.netlib.org/ode/vode.f
         This integrator accepts the following pars in
         set_integrator() method of the ode class:
           atol=float|seq
           rtol=float|seq
           lband=None|int
           rband=None|int
           method='adams'|'bdf'
           with_jacobian=0|1
           nsteps = int
           (first|min|max)_step = float
           order = int        # <=12 for adams, <=5 for bdf
```

---

**Version:**
$Id: ode.py,v 1.2 2003/12/13 13:44:49 pearu Exp $


|  |  |
| --- | --- |
| Classes | [hide private] |

ode  
ode - a generic interface class to numeric integrators.

IntegratorBase

vode


|  |  |
| --- | --- |
| Functions | [hide private] |

|  |  |
| --- | --- |
| noneFn() | source code |

|  |  |
| --- | --- |
| find\_integrator(name) | source code |

|  |  |
| --- | --- |
| test1(f) | source code |

|  |  |
| --- | --- |
| test2(f, jac) | source code |

|  |  |
| --- | --- |
| f1(t, y) | source code |

|  |  |
| --- | --- |
| f2(t, y) | source code |

|  |  |
| --- | --- |
| jac(t, y) | source code |

| Home | Trees | Indices | Help | PyDSTool |
| --- | --- | --- | --- | --- |

|  |  |
| --- | --- |
| Generated by Epydoc 3.0.1 on Fri May 4 15:24:06 2012 | http://epydoc.sourceforge.net |
